# Amylosucrase as a Versatile Biocatalyst for Next‐Generation Functional Ingredients: Starch Modification and Glycoside Synthesis

**DOI:** 10.1111/1541-4337.70656

**Published:** 2026-09-16

**Authors:** Dong‐Ho Seo, Byung‐Hoo Lee, Sang‐Ho Yoo

**Affiliations:** ^1^ Department of Food Science & Biotechnology, and Carbohydrate Bioproduct Research Center Sejong University Seoul Republic of Korea

**Keywords:** amylosucrase, functional carbohydrates, nutraceuticals, starch modification, transglycosylation

## Abstract

Amylosucrase (ASase, E.C. 2.4.1.4) is a transglucosidase that catalyzes α‐glucan synthesis and transglycosylation reactions using sucrose as the sole substrate. Unlike Leloir glycosyltransferases requiring expensive nucleotide sugars, ASases utilize readily available sucrose as a cost‐effective glucose donor, offering economic advantages for industrial applications. This review comprehensively examines the current state of ASase research, covering structural characteristics, catalytic mechanisms, substrate specificities, and industrial applications. Major applications include the synthesis of functional carbohydrates, such as short‐chain glucan microparticles for encapsulation and rare sugars (turanose and trehalulose), as well as starch modification for enhanced nutritional profiles. Furthermore, the enzyme plays a crucial role in generating α‐glycosides of bioactive compounds, significantly improving their physicochemical stability and bioavailability. Recent advances in protein engineering have improved enzyme thermostability and expanded substrate scope, though challenges remain in industrial‐scale implementation. Current limitations in enzyme stability and product yields are critically analyzed, alongside future research directions for improving catalytic performance and process economics. The diverse applications of ASases in food, nutraceutical, and cosmetic industries demonstrate their potential as versatile biocatalysts for producing functional ingredients with improved properties.

## 1 Introduction

Glucansucrases (GSases) typically biosynthesize various α‐glucans with α‐1,6, α‐1,3, and mixed α‐1,3/α‐1,6 linkages using sucrose as the sole substrate. Amylosucrase (ASase, E.C. 2.4.1.4) is unique among GSases in two aspects: it exclusively produces α‐1,4 glucan and is the only member classified within the glycoside hydrolase (GH) family 13 (Seo et al. 2020). The ASase reaction mechanism involves several steps. First, ASase reacts with sucrose, releasing fructose and forming a covalent ASase‐glucosyl intermediate (Jensen et al. 2004). This complex can then undergo a hydrolysis reaction with water's hydroxyl (─OH) group, releasing glucose. In a subsequent step, the glucose intermediate can react with another glucose molecule to form maltose. This maltose then acts as an acceptor molecule for the formation of glucan with α‐1,4 bonds in a non‐processive reaction known as polymerization or transglycosylation (Albenne et al. 2004). When a sufficient concentration of fructose is present, it can also serve as an acceptor, leading to the isomerization reaction that produces sucrose isomers such as turanose and trehalulose (Seo et al. 2020). Fundamentally, ASase catalyzes the transfer of a glucosyl moiety from sucrose to the hydroxyl group of an acceptor molecule, forming a new *O*‐glycosidic bond (Tian et al. 2018). The unique enzymatic properties of ASase make it a versatile tool for producing a wide range of α‐*O*‐glycosidic compounds by using various acceptor molecules, such as flavonoids, isoflavones, and other polyphenols. These diverse ASase products have potential applications in the nutraceuticals, pharmaceuticals, and bio‐industrial sectors (Tian et al. 2018). While previous reviews have provided valuable insights into the fundamental enzymatic properties of ASase (Tian et al. 2018), its microbial discovery (Seo et al. 2020), or general starch modification strategies (Miao and BeMiller 2023), they remain largely descriptive. To clearly differentiate from these prior works and address recent breakthroughs, this review moves beyond a simple literature compilation to offer a critical, forward‐looking synthesis. Specifically, it systematically integrates advanced AI‐driven computational designs—such as protein language models (PLMs) and molecular dynamics (MD) simulations—for predictive enzyme engineering. Furthermore, this review provides a rigorous evaluation of structure‐product specificities and industrial scale‐up bottlenecks, including downstream processing via simulated moving bed (SMB) chromatography, thereby offering comprehensive perspectives for the next generation of industrial implementation.

## 2 Comparative Analysis of Microbial ASases and Enzymatic Properties

**FIGURE 1:**
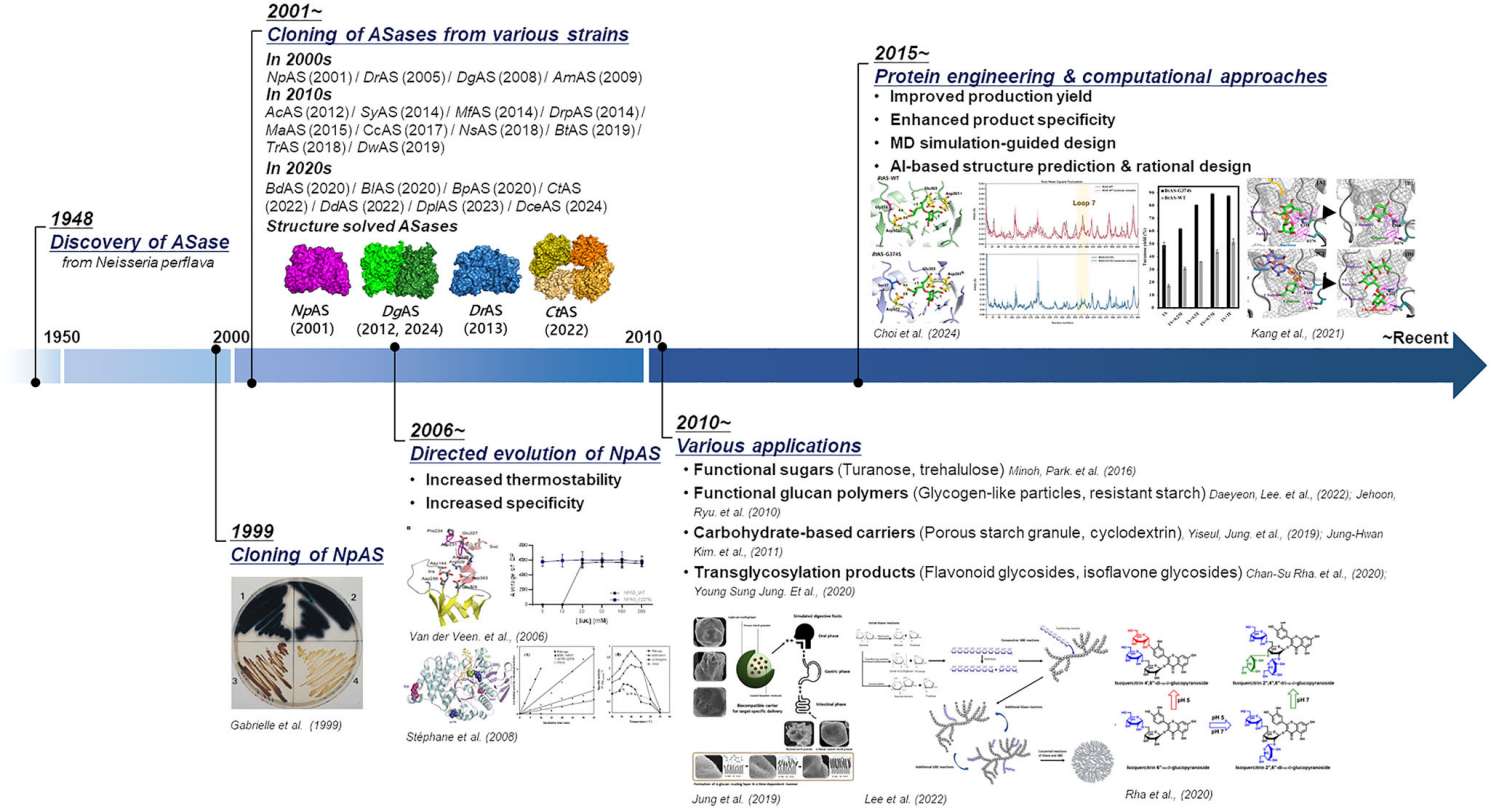
Timeline of amylosucrase (ASase) research milestones. The figure illustrates major developments from discovery (1948) to present, including gene cloning, structural elucidation, protein engineering, industrial applications, and recent computational approaches for rational design.

The existence of ASase was first identified in a strain of *Neisseria perflava* isolated from the meninges in 1946 (Hehre and Hamilton 1946). Subsequent to this discovery, the ASase from *N. polysaccharea* (*Np*AS) was successfully cloned and expressed in *Escherichia coli* in 1997 (Büttcher et al. 1997). Advances in genome sequencing and bioinformatics subsequently enabled the identification of numerous ASase genes across various microbial genomes (Figure 1). However, many of these genes were initially misannotated, often labeled as other members of the GH 13 family, such as α‐amylase, trehalose synthase, or sucrose phosphorylase (Perez‐Cenci and Salerno 2014; Pizzut‐Serin et al. 2005; Seo et al. 2020). To overcome this challenge, researchers began identifying putative ASase genes from various microorganisms based on their amino acid sequence homology to *Np*AS. This approach led to the cloning, expression, and characterization of ASase genes from genera including *Alteromonas*, *Arthrobacter*, *Bifidobacterium*, *Cellulomonas*, *Deinococcus*, *Methylobacillus*, *Methylomicrobium*, and *Synechococcus* in *E. coli* (Seo et al. 2020). Despite this progress, not all genes identified by homology proved to be ASases. For example, the putative ASase gene from *Xanthomonas campestris*, which showed high sequence identity to *Np*AS, was found to be a sucrose hydrolase (SUH) that only hydrolyzes sucrose and does not produce α‐1,4 glucans and sucrose isomers (Champion, Remaud‐Simeon, et al. 2009; H.‐S. Kim et al. 2004). Structural studies later revealed that SUH's exclusive sucrose hydrolysis activity is due to differences in the amino acid sequence of the active pocket surrounding the active site (Champion, Remaud‐Simeon, et al. 2009; M.‐I. Kim et al. 2008).

Beyond sequence identification, characterization studies revealed significant functional diversity among microbial ASases (Table 1). While the optimal pH for ASase activity remains consistent across different bacterial sources (pH 6–8), their optimal temperatures and thermal stabilities vary significantly depending on the origin (Seo et al. 2020). Among various ASases, those from *D. geothermalis* (*Dg*AS) and *Np*AS have been most extensively studied for their enzymatic characteristics, reaction mechanisms, and practical applications. *Np*AS exhibits high polymerization activity but low thermal stability, whereas *Dg*AS has high thermal stability and specific activity but low polymerization activity (Seo et al. 2020). Structural analyses have revealed that these differences are linked to variations in the shape of the active pocket near the active site of *Dg*AS and *Np*AS (Guérin et al. 2012). Other microbial ASases also show distinct characteristics, such as the exceptional thermostability of *Truepera radiovictrix* ASase [melting temperature (*T_m_*) of 69.6°C], and the diverse product specificities among *Bifidobacterium* species ASases, which produce varying sucrose isomer patterns, including species‐specific turanose and trehalulose (Seo et al. 2020). These structural differences among ASases from different microbial sources result in variations in both the product ratios of isomerization reactions and the acceptor specificity for various hydroxylated compounds.

**TABLE 1:** A summary of diverse microbial amylosucrases (ASases) and their enzymatic properties.

Name	GenBank information			Optimal conditions		*T_m_* (°C)	Oligomeric state	References
	Source microorganism	Accession no.	Annotation	Temperature (°C)	pH			
*Ac*AS	*Pseudarthrobacter chlorophenolicus* A6 (formerly *Arthrobacter chlorophenolicus* A6)	ACL41561.1	ɑ‐Amylase catalytic region	45	8	NR	Monomer	Seo et al. (2020)
*Am*AS	*Alteromonas macleodii* KCTC 2957	BAG82876.1	Amylosucrase	45	8	NR	NR	Seo et al. (2020)
*Bd*AS	*Bifidobacterium dentium*	WP_003842696.1	ɑ‐Amylase family protein	35	5	47.8	NR	S.‐Y. Kim, Seo, et al. (2020)
*Bl*AS	*B. longum*	WP_101026183.1	ɑ‐Amylase family protein	50	6	45.0	NR	S.‐Y. Kim, Seo, et al. (2020)
*Bp*AS	*B. pseudocatenulatum*	WP_118284197.1	ɑ‐Amylase family protein	30	8	49.0	NR	S.‐Y. Kim, Seo, et al. (2020)
*Bt*AS	*B. thermophilum*	WP_044279707.1	Amylosucrase	50	6	61.9	NR	S.‐W. Choi et al. (2019)
*Cc*AS	*Cellulomonas carboniz* T26	KGM11272.1	Amylosucrase	40	7	47.8	Monomer	Seo et al. (2020)
*Dce*AS	*Deinococcus cellulosilyticus* KACC 11606	GEM48165.1	ɑ‐Amylase	40	6‐9	47.6	Dimer (pH 8.0) Trimer (pH 6.0)	C.‐Y. Lee et al. (2024)
*Dd*AS	*D. deserti* VCD115	ACO45430.1	Putative amylosucrase	35	7.5	NR	NR	Bae et al. (2022)
*Dg*AS	*D. geothermalis* DSM 11300	ABF44874.1	Amylosucrase	50	8	61.4	Dimer	Seo et al. (2020)
*Dpl*AS	*D. planocerae* KCTC 33809	WP_102128133.1	ɑ‐Amylase family protein	30	7	44.5	NR	Kang et al. (2023)
*Dr*AS	*D. radiodurans*	WP_010887578	ɑ‐Amylase family protein	50	8	NR	Dimer	Seo et al. (2020)
*Drp*AS	*D. radiopugnans*	QCT05769	Amylosucrase	40	8	50.7	Dimer	Seo et al. (2020)
*Dw*AS	*D. wulumuqiensis*	WP_017868998.1	ɑ‐Amylase family protein	45	9	56.2	NR	Seo et al. (2020)
*Ma*AS	*Methylotuvimicrobium alcaliphilum* 20Z (formerly *Methylomicrobium alcaliphilum* 20Z)	CCE22312.1	Putative amylosucrase, ɑ‐amylase	30	8	NR	Monomer	But et al. (2015)
*Mf*AS	*Methylobacillus flagellatus* KT	ABE50875.1	ɑ‐Amylase catalytic region	45	8.5	50.6	Dimer	Seo et al. (2020)
*Np*AS	*Neisseria polysaccharea* ATCC 43768	CAA09772.1	Amylosucrase, partial	35	8	51.5	Monomer	Büttcher et al. (1997)
*Ns*AS	*N. subflava* NJ9703	EFC51554.1	Amylosucrase	45	8	NR	NR	Seo et al. (2020)
*Sy*AS	*Synechococcus* sp. 7002	SMQ77851.1	Amylosucrase	30	6.5	NR	NR	Perez‐Cenci and Salerno (2014)
*Tr*AS	*Truepera radiovictrix *DSM 17093	ADI14158.1	ɑ‐Amylase catalytic region	45	7.5	69.6	Dimer	Zhu et al. (2018)
*Xc*AS	*Xanthomonas campestris* pv. *campestris str*. ATCC 33913	AAM42629.1	Amylosucrase or ɑ‐amylase	30	7.5	NR	NR	C. Yang, Fan, et al. (2019)

Abbreviation: NR, not reported.

The diverse biochemical characteristics and reaction profiles of microbial ASases summarized in this section are fundamentally governed by their unique structural architectures. While the foundational structural biology of these enzymes has been comprehensively reviewed in previous literature (Tian et al. 2018), understanding how specific molecular determinants dictate these functional variations is crucial for advanced biocatalyst design. Therefore, the detailed mechanistic basis linking these macroscopic biochemical properties to specific structural elements, such as loop flexibility and active site topology, is systematically explored in Section 3.

## 3 Structural Determinants of Catalytic Specificity and Stability

An understanding of the tertiary structure of an enzyme is imperative for elucidating its reaction mechanism (Selvaraj et al. 2022). Among the various ASases, the tertiary structure of *Ct*AS (ASase from *Calidithermus timidus*), *Dg*AS, *Dr*AS (ASase from *D. radiodurans*) and *Np*AS have been solved (Guérin et al. 2012; Mirza et al. 2001; Oh et al. 2025; Skov et al. 2001, 2013; Tian et al. 2022). Although the overall backbone structure remains constant, it has been determined that the ASase properties exhibit variability due to variations in certain residues surrounding the active pocket (Guérin et al. 2012; Skov et al. 2001, 2013). ASase possesses the N, A, B, and C domains characteristic of the GH13 family, along with an additional B′ domain that contributes to its unique enzymatic properties (Jensen et al. 2004).

The N domain of ASase has been demonstrated to exert a significant influence on its oligomeric state (Oh et al. 2025). While the oligomeric state of *Np*AS exists as a monomer, *Dg*AS and *Dr*AS appear as a dimer (Roblin et al. 2013). A previous study indicated that the dimeric state of *Dg*AS contributes to its higher thermostability compared to the monomeric *Np*AS (Guérin et al. 2012). A recent study demonstrated that substituting a single residue in the N‐domain of DgAS (R30A) caused the typically dimeric protein to exist as a monomer, resulting in slightly decreased thermal stability and melting temperature (Oh et al. 2025). Interestingly, the *Ct*AS was shown to have a homotetrameric quaternary structure, a feature not previously observed in other ASases (Tian, Xu, et al. 2019). It has been established that the structure consists of both interdimeric and intradimeric interfaces. The intradimeric interfaces of *Ct*AS appear to be analogous to those of *Dg*AS and *Dr*AS (Tian et al. 2022). The distinctive interdimeric interface of *Ct*AS is stabilized by hydrophobic interactions and hydrogen bonds. Protein engineering study has demonstrated that specific mutations (L382P, S414N, P618I, and H631R) at the interdimeric interface can enhance these stabilizing interactions, thereby increasing thermostability (Tian et al. 2022). The findings indicate that the oligomeric state of ASases exerts a substantial influence on their thermostability. However, the relationship between oligomeric state and thermostability appears to be intricate and may encompass additional structural factors beyond quaternary structure.

**FIGURE 2:**
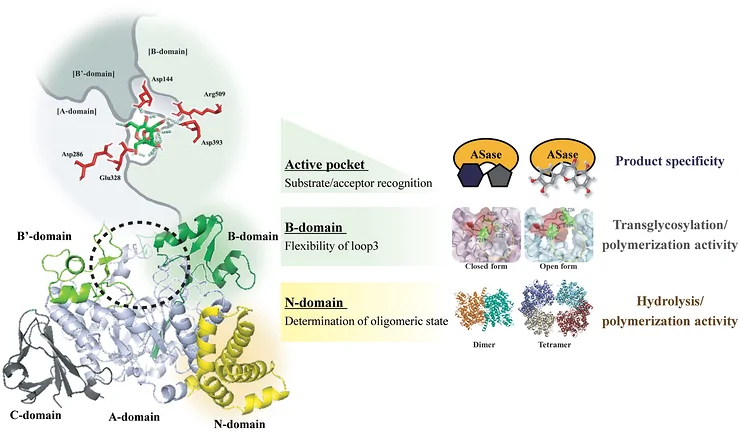
Structural organization and functional domains of amylosucrase (ASase). The three‐dimensional structure of ASase shows the characteristic domain organization of the GH13 family, including the N‐domain (blue), A‐domain with (β/α)8‐barrel structure (green), B‐domain (yellow), B′‐domain (orange), and C‐domain (purple). The active site is located at the interface between domains A, B, and B′, with catalytic residues (aspartate nucleophile and glutamate acid/base) positioned at subsites −1 and +1. The acceptor binding subsites (+1 to +4) accommodate various substrate molecules. The reaction mechanism involves formation of a covalent glucosyl‐enzyme intermediate followed by transfer to acceptor molecules, enabling diverse product formation including transglycosylation products, polymers with varying chain lengths, and hydrolysis products. The oligomeric state and loop flexibility significantly influence substrate binding and product specificity, determining the balance between polymerization, transglycosylation, and hydrolysis activities.

The B and B′ domains, in conjunction with a segment of the A domain, constitute a unique active pocket within ASase: The A domain forms the characteristic (β/α)_8_‐barrel of the GH13 family, with the B and B′ domains inserted within the A domain's primary amino acid sequence. As a tertiary structure, the B and B′ domains influence substrate binding (Figure 2). The catalytic sites of the ASase have been identified as an aspartate acting as a catalytic nucleophile and a glutamate functioning as a general acid/base within the active pocket of the enzyme (Skov et al. 2001). The active site contains multiple subsites for substrate recognition, designated as −1, +1, +2, +3, and +4 subsites. The −1 subsite accommodates the glucosyl donor moiety of sucrose, while the +1 to +4 subsites are responsible for acceptor substrate binding (Albenne et al. 2004). The reaction mechanism of ASase follows a double displacement reaction involving two catalytic residues. First, when the glucosyl moiety of sucrose binds at the −1 subsite, an aspartate residue acting as a catalytic nucleophile attacks the C_1_ carbon of glucose, while a glutamate residue functions as a general acid to protonate the leaving fructose. This results in the formation of a covalent β‐glucosyl‐enzyme intermediate. In the second step, when an acceptor molecule with a hydroxyl group is positioned at the +1 subsites, the glutamate residue acts as a general base to activate the acceptor's hydroxyl group, facilitating nucleophilic attack on the glucosyl‐enzyme intermediate and resulting in product formation with the release of the enzyme (Jensen et al. 2004). Numerous studies have demonstrated that alterations within the +1 to +4 subsites significantly influence ASase product specificity. In the *Np*AS, the substitution of Asp394, which corresponds to +2 and +3 subsites, with the bulkier Glu394 resulted in reduced α‐1,4 glucan chain elongation compared to the wild‐type enzyme. This reduction can be attributed to steric hindrance that impairs acceptor binding (Schneider et al. 2009). Furthermore, the substitution of Arg415 in the +4 subsite with alanine enhanced oligosaccharide elongation (Albenne et al. 2004). Moreover, the mutation of Arg226 in the +2 subsite to Asn resulted in a substantial increase in the rate of glycosylation, due to optimizing the positioning of the acceptor's glucosyl moiety for catalysis (Cambon et al. 2014). In addition, the enzymatic properties of ASase are modulated by conformational changes associated with internal loop flexibility. In *Dg*AS, the reduced flexibility of loop3 in the B domain enhances acceptor affinity, leading to enhanced transglycosylation and polymerization activities (Hong et al. 2020; Seo et al. 2019). These flexible loops also facilitate the binding of long‐branched glucans to the ASase surface, mediating reactions between α‐glucans (such as starch and glycogen) and acceptor molecules (Albenne et al. 2007). Consequently, the composition of amino acid residues in the active pocket and the flexibility of surrounding loops have been demonstrated to significantly influence ASase activities.

Recent studies have also revealed that the oligomeric state of ASases significantly influences their polymerization activity. Structural analysis has demonstrated that, upon transitioning from its native dimeric form to a monomeric state (as observed in the R30A mutant), *Dg*AS experiences a substantial decrease in surface area available for oligosaccharide binding (Oh et al. 2025). This reduction in binding surface area has been shown to result in diminished interaction with longer‐chain maltooligosaccharides, consequently leading to increased production of shorter degree of polymerization (DP) α‐glucans. The oligomeric state of ASase from *D. cellulosilyticus* (*Dce*AS) exhibited a pH‐dependent transitions, existing as a trimer at pH 6 and a dimer at pH 8. The pH‐dependent oligomerization directly influenced the catalytic properties of the enzyme (C.‐Y. Lee et al. 2024). The trimeric form at pH 6 exhibited enhanced polymerization activity and synthesized longer‐chain glucans, while the dimeric form at pH 8 showed higher hydrolysis activity and produced shorter‐chain glucans. The authors suggested that the enhanced polymerization activity of the trimer may be attributed to its larger substrate binding surface area. Although extensive structural and functional insights into ASases have been obtained, the precise role of the C‐domain in enzyme function and oligomerization remains to be elucidated. Consequently, the C‐domain represents an unexplored target for rational engineering that warrants further structural investigation.

Collectively, the variations in catalytic and structural properties summarized in Table 1 serve as a strategic roadmap for rational biocatalyst selection. For high‐temperature industrial processes, thermostable variants (e.g., *Dg*AS, and *Tr*AS) are superior candidates not only for their robust operational stability but also for their kinetic advantages in high‐temperature environments, where reduced substrate viscosity facilitates improved mass transfer and minimizes microbial contamination risks. Conversely, the acidic pH optima (pH 5–6) of *Bifidobacterium*‐derived ASases are uniquely suited for the biotransformation of acidic food matrices, such as fruit juices and fermented dairy products, where they can function directly without the need for pH neutralization—a step that often compromises product authenticity and processing costs. Furthermore, pH‐dependent oligomerization strategies allow for tuning the product spectrum, enabling engineers to shift the reaction flux between hydrolysis‐dominated and polymerization‐dominated modes depending on the target functional ingredient. Therefore, rather than selecting enzymes based on single‐parameter optimality, an integrated approach that harmonizes the enzyme's operational window with the specific requirements of the food matrix and process conditions is vital for the rational design of tailor‐made biocatalysts.

## 4 Advanced Computational Design and Protein Engineering of ASases

Naturally occurring ASases frequently lack the robust thermostability and optimal catalytic efficiency required for demanding large‐scale bioprocesses. The *Np*AS exhibits strong polymerization activity at its optimum of 30°C but suffers from rapid thermal deactivation at elevated temperatures, with its half‐life plummeting to approximately 15 min at 50°C (Guérin et al. 2012). Generating random mutant libraries and applying laborious screening enabled the isolation of *Np*AS variants with significantly increased half‐lives at 50°C (Emond et al. 2008). The *Dg*AS inherently possesses exceptional thermostability at 50°C but demonstrates significantly lower polymerization efficiency than *Np*AS (Seo et al. 2020). Semi‐rational mutagenesis focusing on the highly flexible loop 3 within the B domain yielded the *Dg*AS‐A226N variant, which displayed a dramatically improved polymerization to hydrolysis ratio (Seo et al. 2019). Structural and kinetic analyses revealed that this mutation reduced the local flexibility of the loop, stabilizing the substrate‐binding pocket and enabling the enzyme to synthesize longer α‐1,4‐glucan chains efficiently (Seo et al. 2016, 2019). Several high‐throughput colorimetric and fluorometric assays for ASase have emerged to monitor sucrose consumption and transglycosylation activity (Emond et al. 2007; Seo et al. 2019). These quantitative methods successfully indicate overall enzyme turnover but inherently lack the structural resolution required to identify specific target glycosides or detailed product profiles. Traditional chromatographic techniques required for precise product profiling consequently demand extensive labor and time, severely limiting the rapid evaluation of massive mutant libraries (Champion, André, et al. 2009; Emond et al. 2007). Emerging computational technologies and artificial intelligence (AI) provide effective alternative strategies to overcome these conventional screening bottlenecks. The strategic integration of machine learning models offers a compelling approach to bypass these throughput limitations, such as the exorbitant time and resource costs associated with exhaustive experimental screening (K. K. Yang, Wu, et al. 2019). PLMs represent a particularly powerful methodology for next‐generation enzyme design (Ferruz and Höcker 2022; Madani et al. 2023). These advanced AI architectures evaluate vast evolutionary protein sequences to predict thermostabilizing mutations or optimal active site configurations without requiring massive experimental datasets (Biswas et al. 2021; Blaabjerg et al. 2023). Targeted machine learning frameworks have already successfully guided the thermostabilization of homologous GH13 enzymes, such as industrially relevant α‐amylases, by accurately forecasting these stabilizing mutational hotspots (Liao et al. 2024). PLMs similarly enhanced the transglycosylation‐to‐hydrolysis ratio of cyclodextrinase, sharing the identical GH13 family classification with ASases, while requiring the physical screening of fewer than a hundred variants (Nie et al. 2025). Crucially, the translation of these sequence‐based predictions into structural insights has been revolutionized by AI‐driven structure prediction tools, most notably AlphaFold2 and PLM‐based algorithms like ESMFold (Jumper et al. 2021; Lin et al. 2023). Traditionally, rational design and structural analyses were strictly constrained by the availability of experimentally determined x‐ray crystal structures. These advanced predictive models now rapidly generate highly accurate 3D architectures for novel ASases and their variants, effectively bridging the gap between sequence discovery and three‐dimensional mapping. Advanced MD simulations concurrently overcome the limitations of static structural data by providing continuous temporal insights into active site flexibilities. Computational investigations of ASases successfully employed these dynamic models to map the intricate conformational movements of active site loops during catalysis. These simulated trajectories explicitly correlated the local structural flexibility of specific regions, such as Loop 3 and Loop 7, with the resulting transglycosylation efficiency (Hong et al. 2020; S.‐J. Ryu et al. 2024). MD simulations of the engineered *Dg*AS‐I221V variant specifically demonstrated that altered flexibility around Loop 3 and Loop 7 directly enlarges the active site pocket volume (S.‐J. Ryu et al. 2024). This computationally verified structural expansion enabled the enzyme to achieve higher transglycosylation efficiencies with bulky flavonoid acceptors, such as naringenin, by lowering the steric hindrance during initial substrate binding. Computational investigations of the *Dg*AS‐A226N mutant similarly utilized dynamic simulations to explain its enhanced reactivity toward small phenolic compounds. These temporal models revealed that the specific asparagine substitution geometrically stabilized the active conformation of hydroquinone within the binding pocket, explicitly leading to a 1.4‐fold increase in experimental α‐arbutin production (Hong et al. 2020). These successful translations of computational insights into functional variants establish a concrete engineering blueprint: precisely tuning the flexibility of Loop 3 and Loop 7 through targeted mutagenesis at their hinge regions can systematically expand the acceptor promiscuity of ASases for various bulky industrial substrates. A comprehensive summary of these structural engineering strategies, correlating computational predictions with experimentally validated functional outcomes across various ASases, is provided in Table 2. Integrating these combined data‐driven workflows will dramatically accelerate the design of tailor‐made ASase biocatalysts, shifting the engineering paradigm from trial‐and‐error to rational, predictive generation.

**TABLE 2:** Comprehensive summary of structural engineering strategies and their functional outcomes in diverse amylosucrases (ASases).

Enzymes/Variants	Target region	Computational and structural analysis	Experimental validation (functional outcome)	References
*Dg*AS‐B (chimeric)	Entire B domain	MD simulations suggested that the active‐site conformation became more rigid following B domain replacement.	kcat​/Km​ increased 1.9‐fold (acceptor) and 1.6‐fold (donor) towards 4‐methylumbelliferone (MU) compared to WT.	Seo et al. (2016)
*Dg*AS‐A226N	Loop 3	MD simulations showed A226N stabilized the localized structure near the phenolic‐compound binding site.	Sucrose affinity increased 2.0‐fold; overall α‐arbutin production increased 1.4‐fold.	Hong et al. (2020)
*Dg*AS‐I221V	Loop 3 and 7 (Subsite +1)	Altered flexibility of loop 3/7 significantly expanded the active‐site pocket volume, reducing steric hindrance.	Transglycosylation efficiency (kcat​/Km​) towards naringenin increased 5.15‐fold.	S.‐J. Ryu et al. (2024)
*Np*AS‐F417A	Subsite −1 & active‐site loops	Combined x‐ray/MD revealed local rigidity at the −1 subsite coexisting with flexibility in active‐site‐shaping loops.	kcat​ increased 98‐fold in the presence of glycogen.	Albenne et al. (2007)
*Np*AS‐A289P/F290L	Active‐site vicinal residues	x‐Ray structures supported by MD demonstrated parallel structural dynamics, revealing a stabilized −1 subsite interacting with dynamic topological loops.	Specificity enhanced toward both donor and acceptor compared to WT.	Champion et al. (2012)
*Np*AS‐9 combined	Active site (9 distinct mutations)	x‐Ray crystallography and MD explicitly explained the structural origins of its elevated and highly controlled activity.	Hydrolysis activity increased 5.6‐fold; achieved narrow product distribution of soluble malto‐oligosaccharides (DP 2–21).	Vergès et al. (2017)
*Np*AS Subsite −1 variants (Y147X, H187X, F250X, R284X, H392X, R509X)	Subsite −1 hydrogen‐bond network	FoldX‐based stability‐change (ΔΔ*G*) predictions guided mutation selection (without MD trajectory analysis).	Subsite −1 mutations altered thermodynamic stability and catalytic activity via hydrogen‐bond reorganization.	Daudé et al. (2013)
*Drp*AS‐Q299K / Q299R	Residue 299 (Acceptor binding)	In silico docking demonstrated that basic substitutions (e.g., Lys299) geometrically favored rutin accommodation.	Rutin transglycosylation increased 10‐fold (Q299K) and 4‐fold (Q299R) compared to WT.	Seo et al. (2020)
*Dw*AS‐V217I (reversal mutant)	Residue 217 (DgAS I221 equivalent)	Docking revealed *Dw*AS naturally exhibits lower steric hindrance for naringenin due to Val217. The V217I reversal mutant experimentally validated this structural hypothesis.	Introduction of steric bulk (V217I) precipitously decreased transglycosylation efficiency (kcat​/Km​) by 179‐fold.	S.‐J. Ryu et al. (2024)

Leveraging these engineered biocatalysts, the subsequent industrial applications of ASases can be progressively categorized based on the complexity of the acceptor systems—ranging from de novo polymerization and the synthesis of rare sugars (e.g., sucrose isomers) (Section 5) to macromolecular starch primers (Section 6) and the diverse structural landscape of bioactive glycosides (Section 7).

## 5 Bioprocess Strategies for Functional Carbohydrate Synthesis

### 5.1 Self‐Assembly Mechanisms and Encapsulation Efficiency of SCG Microparticles

**FIGURE 3:**
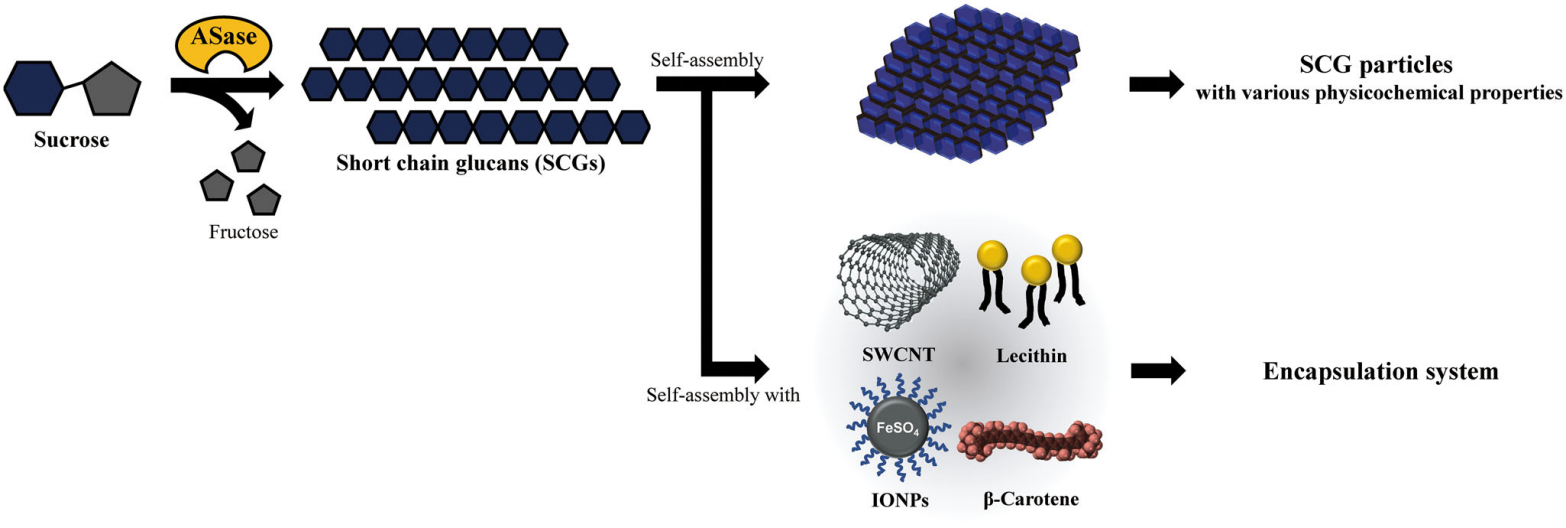
Short‐chain glucan (SCG) particle formation and encapsulation applications. ASase catalyzes the synthesis of short‐chain glucans from sucrose through a non‐processive mechanism, producing glucose polymers with varying degrees of polymerization (DP) linked by α‐1,4 glycosidic bonds. The resulting SCGs spontaneously self‐assemble into stable microparticles under equilibrium conditions. These SCG particles serve as versatile encapsulation systems for various functional materials including single‐walled carbon nanotubes (SWNTs), lecithin for surface stabilization, iron oxide nanoparticles (IONPs) for magnetic applications, and β‐carotene for enhanced bioavailability. The encapsulation process improves the stability, dispersibility, and functional properties of the incorporated materials, enabling applications in food matrices, immunomagnetic separation systems, and delivery of bioactive compounds with enhanced resistance to environmental degradation.

ASase catalyzes the synthesis of short‐chain glucan (SCG) from sucrose through its unique transglycosylation (or polymerization) activity. These SCGs are composed of glucose units linked by α‐1,4 glycosidic bonds, with varying DPs depending on reaction conditions (S.‐Y. Kim, Seo, et al. 2020; Seo et al. 2020). The non‐processive nature of the reaction mechanism results in the formation of a heterogeneous mixture of oligosaccharides. Under equilibrium conditions, the SCG biosynthesized by ASase spontaneously self‐assembles into stable, structurally well‐defined particles with micrometer dimensions (Figure 3) (Luo et al. 2023). Several studies have demonstrated the potential of SCG particles as encapsulation systems for various materials. Lim et al. (2014) first reported that SCGs produced by *Dg*AS could encapsulate single‐walled carbon nanotubes through a self‐assembly process, resulting in hybrid SCG‐nanotube particles. To improve SCG particle stability in various applications, lecithin has been employed as a surface stabilizer to prevent premature aggregation during particle nucleation. This amphiphilic molecule acts as a steric stabilizer, maintaining the colloidal stability of ASase‐produced SCG in aqueous solution (Letona et al. 2019). Furthermore, metallic nanoparticles can function as nucleation seeds during ASase‐mediated SCG particle formation, enabling their application as effective biomagnetic isolation systems (Luo et al. 2023). In subsequent study, β‐carotene, an antioxidant characterized by low water solubility and poor chemical stability, was successfully encapsulated within ASase‐synthesized SCG particles. The encapsulation process enhanced both the chemical stability and potential bioavailability of β‐carotene, resulting in microparticles that exhibited superior resistance to UV light and chemical oxidation compared to free β‐carotene or its nanodispersion form (Letona et al. 2017).

A recent study investigated the synthesis of SCG particles using four distinct ASases from *Bifidobacterium* species, revealing significant variations in their physicochemical properties. These variations encompassed parameters such as particle morphology, size, crystallinity, and iodine binding capacity (Y.‐J. Kim et al. 2024). The SCG particles exhibited different characteristics depending on the enzyme used, with production yields ranging from 39.8% to 63.2% and particle size from 0.92 to 3.17 µm. Notably, differences in the DP distribution of linear glucans were observed, with number‐average DPs (DPn) ranging from 18.6 to 25.8. These enzyme‐specific variations in both chain length distribution and reaction kinetics directly influenced the physicochemical properties of the resulting SCG particles. The finding demonstrated that the selection of specific ASase types could serve as a strategic tool for controlling and customizing SCG particle properties for various applications.

While these pioneering studies collectively highlight the versatility of SCG microparticles, a critical synthesis of the literature reveals that encapsulation efficiency, particle size stability, and optimal storage conditions vary substantially depending on the nature of the core material. For example, encapsulating highly lipophilic molecules like β‐carotene necessitates amphiphilic stabilizers (e.g., lecithin) to prevent premature aggregation and maintain colloidal stability over time. In contrast, rigid matrices such as carbon nanotubes or metallic nanoparticles act as direct nucleation seeds, inherently altering the self‐assembly kinetics and final particle dimensions. Currently, the field lacks standardized metrics for reporting these parameters across diverse hybrid systems. To transition from proof‐of‐concept to commercial delivery systems, future studies must systematically correlate the physicochemical properties of the target payload with the resulting SCG encapsulation efficiency and long‐term stability under standardized storage conditions.

### 5.2 Optimization of Isomerization Yields for Turanose and Trehalulose Production

Sucrose isomers, which feature different glycosidic linkages between glucose and fructose units compared to conventional sucrose (α‐D‐glucopyranosyl‐(1→2)‐β‐D‐fructofuranose), have emerged as promising industrial alternatives due to their beneficial properties such as reduced glycemic response, lower cariogenic potential, and improved processing stability (Tian, Deng, et al. 2019). ASase can synthesize sucrose isomers, including turanose (α‐D‐glucopyranosyl‐(1→3)‐α‐D‐fructofuranose) and trehalulose (α‐D‐glucopyranosyl‐(1→1)‐α‐D‐fructopyranose), through isomerization reactions utilizing fructose released from sucrose as an acceptor substrate (Seo et al. 2020).

Turanose has garnered particular interest for industrial applications due to its unique stability under acidic conditions, making it suitable for use in fruit juices and yogurt drinks (Han et al. 2021). While turanose is present in natural honey, its limited availability in natural sources has led to the development of ASase‐based enzymatic synthesis as an efficient alternative for industrial production. R. Wang et al. (2012) reported efficient turanose production using *Np*AS, achieving a yield of 56.2% under optimized conditions (2.5 M sucrose, 400 U/L *Np*AS, 35°C, and 120 h). In subsequent studies, researchers focused on identifying ASases with enhanced thermostability and turanose biosynthetic efficiency to overcome the limitations of *Np*AS. For instance, ASase from *N. subflava* (*Ns*AS) demonstrated improved performance over *Np*AS, achieving a turanose yield of 76% under optimal conditions (1.5 M sucrose, 1 M fructose, 40°C, pH 8.0, and 120 h) (M.‐O. Park et al. 2019). Similarly, *Bt*AS achieved a 43.3% turanose yield under specific conditions, with the yield showing an inverse correlation with reaction temperature and a strong dependence on fructose concentration (S.‐W. Choi et al. 2019). Subsequent efforts to improve turanose production focused on enzyme engineering and novel enzyme discovery. While ASase identified from thermal aquatic metagenome exhibited turanose yields comparable to *Np*AS with enhanced thermostability (Agarwal et al. 2019), structure‐guided mutagenesis of existing ASases proved more effective. The *Np*AS G396S variant, in which glycine at the +2 subsite was substituted with serine, displayed reduced polymerization and enhanced isomerization activity, achieving 523 g/L turanose (76.5% conversion yield) using 2.5 M sucrose and 0.75 M fructose (Su et al. 2020). Similarly, structure‐based engineering to *Bt*AS by substituting key residues with corresponding *Np*AS residues significantly improved performance. The *Bt*AS Y414F/P200R double mutant reached 89.3% turanose yield (Jun et al. 2022), while the *Bt*AS G374S variant achieved 592.9 g/L turanose production. To enhance industrial applicability, *Bt*AS G374S was immobilized on CPC silica carriers, resulting in improved operational stability with a half‐life of approximately 50 h at 55°C. The immobilized enzyme retained 68% of its initial activity after 10 reuse cycles (B.‐Y. Choi, Seo, et al. 2024).

Trehalulose, a naturally occurring sugar found in stingless bee honey, has gained attention as a functional sweetener due to its low glycemic response and antioxidant activity (Seevanathan et al. 2024). Its high solubility makes it particularly suitable for liquid food applications and highly sweetened products. Sucrose isomerase primarily produces isomaltulose, with trehalulose as a minor byproduct (Salvucci 2003). Other enzymes such as trehalose synthase, maltases, and α‐glucosidases can also catalyze trehalulose formation, but generally with low yields (Ernits et al. 2021; Garcia‐Gonzalez et al. 2023; Liang et al. 2013). Among these enzymatic approaches, ASase have emerged as a promising catalyst for trehalulose production. While most ASases predominantly catalyze turanose formation, ASases from *Deinococcus* species exhibit preferential selectivity toward trehalulose biosynthesis (Seo et al. 2020). Structural analysis revealed that this selectivity stems from differences in active site architecture. *Np*AS contains a key amino acid residue that anchors the O3′ hydroxyl of fructose, favoring turanose formation, whereas *Dg*AS lacks this anchoring residue, resulting in enhanced trehalulose formation (Guérin et al. 2012). Similarly, *Dr*AS displays distinct structural features, including unique loop conformation near the active site and alternative fructose binding mode, contributing to its higher trehalulose production efficiency (Pizzut‐Serin et al. 2005; Skov et al. 2013). ASase from *D. deserti* (*Dd*AS) exhibited a trehalulose‐to‐turanose ratio of 2:1, achieving 24.6% trehalulose yield with 2 M sucrose at 35°C for 120 h. The addition of fructose as an acceptor molecule significantly enhanced production, with *Dd*AS reaching a maximum trehalulose yield of 36% under optimized conditions (2 M sucrose and 0.75 M fructose), corresponding to a productivity of 1.94 g/L/h (Table 3) (Bae et al. 2022).

**TABLE 3:** Turanose and trehalulose production by different amylosucrases (ASases).

ASase	Enzyme dosage (U^a^/L)	Volumetric productivity^b^ (g/L/h)	Selectivity^c^ (%)	Substrate conversion (%)	Yield (%)	Concentration (g/L)	Reaction conditions				Reference
							Sucrose (M)	Fructose (M)	Temperature (°C)	Time (h)	
Production of turanose											
*Np*AS	400	4.01	NR	100	56.2	480.9	2.5	—	35	120	R. Wang et al. (2012)
*Np*AS G396S	NR	10.91	NR	NR	76.5	523.7	2.0	0.5	30	48	Su et al. (2020)
*Ns*AS	400	3.25	NR	NR	76.0	390.2	1.5	1.0	40	120	M.‐O. Park et al. (2019)
*Bt*AS	400	3.09	NR	NR	43.3	148.2	1.0	1.0	50	48	S.‐W. Choi et al. (2019)
*Bt*AS Y414F/P200R	400	50.95	NR	NR	89.3	305.7	1.0	1.0	50	12	Jun et al. (2022)
*Bt*AS G374S	1,600	6.18	NR	NR	86.6	592.9	2.0	1.0	50	96	B.‐Y. Choi, Seo, et al. (2024)
*Bp*AS	400	1.41	64.5	100	33.0	67.8	0.6	—	30	132	S.‐Y. Kim, Seo, et al. (2020)
*Bd*AS	400	0.57	62.1	100	36.7	75.4	0.6	—	35	48	S.‐Y. Kim, Seo, et al. (2020)
Production of trehalulose											
*Dd*AS	400	1.40	67.0	100	24.6	168.4	2.0	—	35	120	Bae et al. (2022)
*Dd*AS	400	1.94	60.9	NR	36.3	248.5	2.0	0.75	35	120	

*Note*: “—” means no exogenous fructose added to the reaction mixture.

Abbreviations: ASase, amylosucrase; *Bd*AS, *Bifidobacterium dentium* ASase; *Bp*AS, *Bifidobacterium pseudocatenulatum* ASase; *Bt*AS, *Bifidobacterium thermophilum* ASase; *Dd*AS, *Deinococcus deserti* ASase; *Np*AS, *Neisseria polysaccharea* ASase; NR, not reported; *Ns*AS, *Neisseria subflava* ASase.

^a^ One unit (U) of enzyme activity was defined as the amount of enzyme releasing 1 µmol of reducing sugars, such as fructose, glucose, and sucrose isomers, per minute.

^b^ Volumetric productivity (g/L/h) was calculated by dividing the final concentration of the target product (turanose or trehalulose, g/L) by the total reaction time (h).

^c^ Selectivity (%) was defined as the proportion of the target sucrose isomer among all synthesized sucrose isomers, calculated as the target isomer yield divided by the total isomerization yield × 100.

While the synthesis of rare sugars (e.g., turanose) and SCGs holds significant potential, the current technological maturity is moderate. The primary industrial bottleneck lies not in the enzymatic conversion efficiency, but in the downstream processing; specifically, the separation of isomer mixtures requires high‐cost chromatographic techniques like SMB. For future industrial scaling, research must shift from simple yield optimization to integrated bioprocess design, focusing on minimizing substrate residue and enhancing product purity at scale.

## 6 Enzymatic Modification of Starch: From Structure to Function

Starch, a primary energy source in human nutrition, can be classified into three categories based on digestibility: rapidly digestible starch (RDS), slowly digestible starch (SDS), or resistant starch (RS), each with distinct characteristics (Englyst and Hudson 1996). RDS undergoes enzymatic digestion within 20 min, causing sharp increases in blood glucose levels that may contribute to metabolic disturbances (Gibson et al. 2013). In contrast, SDS is gradually digested over 20–120 min, providing sustained glucose release, while RS resists digestion in the small intestine and reaches the colon intact, where it serves as a substrate for gut microbiota and functions as dietary fiber.

Beyond nutritional concerns, native starch exhibits limitations in physicochemical properties, particularly thermal stability and paste behavior, which restrict its industrial applications (Rashwan et al. 2024). These limitations can be addressed through various modification techniques, including physical, chemical, and enzymatic approaches (Compart et al. 2023). Among enzymatic modification strategies, ASase offers a unique approach by catalyzing the transfer of glucose units to the non‐reducing ends of amylopectin branches, resulting in extended branch length. This structural modification increases the proportions of SDS and RS while reducing RDS content, thereby improving both the nutritional profiles and functional properties of starch.

### 6.1 Mechanism of Branch Chain Elongation and Crystalline Structure Modulation

**FIGURE 4:**
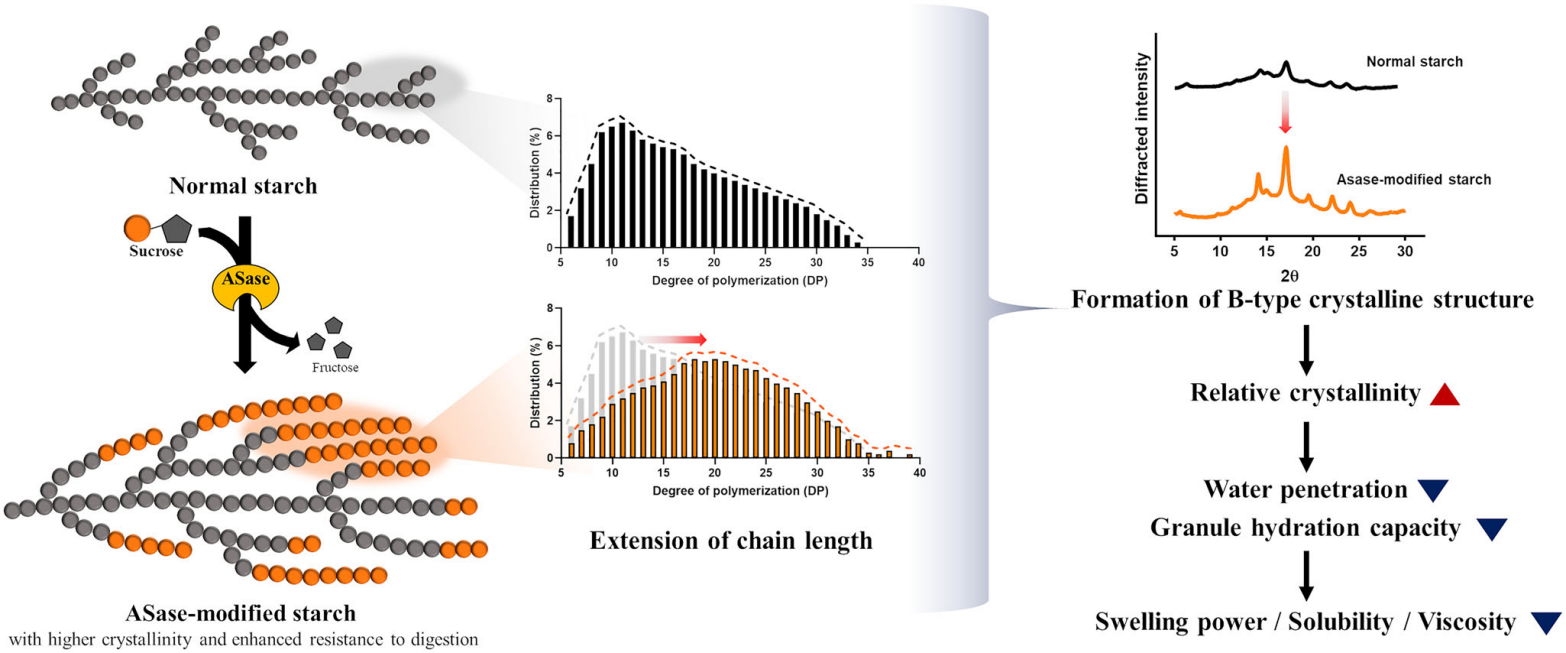
Mechanism of starch modification by amylosucrase (ASase). ASase catalyzes the transfer of glucose from sucrose to the non‐reducing ends of amylopectin branches, resulting in chain elongation. The modification shifts the chain length distribution from predominantly short chains to longer chains. The explicit mechanistic flowchart illustrates how this structural chain extension translates into altered crystalline structures, restricted swelling behaviors, reduced digestibility (formation of resistant starch), and subsequent improvements in macroscopic food functionality.

Building upon the fundamental α‐1,4‐glucan polymerization mechanism that generates SCGs (as discussed in Section 5.1), ASase can also utilize complex macromolecular primers. Rather than initiating de novo synthesis, the enzyme effectively catalyzes the transfer of D‐glucose residues from sucrose to the non‐reducing ends of existing amylopectin (AP) branches (Rolland‐Sabaté et al. 2014). This highly branched structure provides numerous accessible non‐reducing ends due to its branched structure with α‐1,6 linkages, making it an ideal acceptor for ASase. When starch serves as a primer, ASase preferentially elongates existing chains rather than initiating de novo polymer synthesis, resulting in significantly higher reaction efficiency (Miao and BeMiller 2023). This primer‐dependent mechanism directs glucosyl units toward chain elongation rather than side reactions such as sucrose hydrolysis or isomerization, ensuring efficient utilization of sucrose for starch modification. ASase treatment systematically alters the chain length distribution of AP by increasing longer chains (DP 25–36) while decreasing shorter chains (DP 6–12) (Figure 4) (Miao and BeMiller 2023). The extended chains facilitate stable double helix formation, which subsequently align to form B‐type crystalline structures similar to amylose retrogradation patterns (Dominguez‐Ayala et al. 2023; D.‐H. Jung et al. 2022; Miao and BeMiller 2023). Reaction temperature significantly impacts the effectiveness of ASase‐mediated starch modification. D.‐H. Jung et al. (2022) demonstrated that *Dg*AS activity at 30°C produced a considerably higher proportion of side chains with DP > 25 compared to 50°C, leading to enhanced B‐type crystalline structure formation. Lower temperatures resulted in improved thermal stability and substantially higher RS content, nearly doubling that of native starch.

### 6.2 Impact of Structural Modification on Physicochemical Functionality and Food Applications

ASase modification alters the physicochemical properties of starch through chain elongation of AP branches, enhancing its functional characteristics for food application. The elongated branch chains enhance thermal stability, as evidenced by increased gelatinization temperatures and melting enthalpy values across various starch sources (D.‐H. Jung et al. 2022; B. K. Kim, Kim, et al. 2014; I. Park and Mannaa 2024; H. Zhang et al. 2017). These thermal improvements reflect the formation of more stable crystalline structures created by elongated branch chains, which require greater energy input for structural disruption. The melting enthalpy also increases, indicating enhanced crystalline order and stability (B. K. Kim, Kim, et al. 2014; I. Park and Mannaa 2024). As discussed in Section 5.1, the modification promotes B‐type crystalline structure formation, with relative crystallinity increasing in extensively modified starches (R. Wang et al. 2023; H. Zhang et al. 2017). x‐Ray diffraction analysis confirms the formation of B‐type polymorphs characterized by new peaks at 2*θ* = 5°–6° and 17°, contributing to improved heat resistance suitable for food processing applications (B.‐S. Kim et al. 2013; H. Zhang et al. 2017).

ASase treatment significantly affects pasting behavior, with modified starches displaying higher pasting onset temperatures and reduced peak viscosity due to restricted granule swelling (B. K. Kim, Kim, et al. 2014; E. J. Kim et al. 2016; I. Park and Mannaa 2024). Rapid Visco Analyzer analysis reveals increased pasting onset temperatures in waxy corn starch, while peak viscosity decreases substantially (D.‐H. Jung et al. 2022; I. Park and Mannaa 2024). The enhanced crystalline architecture reduces swelling power and solubility across various starch sources (D.‐H. Jung et al. 2022; B. K. Kim, Kim, et al. 2014; E. J. Kim et al. 2016; I. Park and Mannaa 2024; H. Zhang et al. 2017). This reduction occurs because elongated external chains strengthen both amorphous and crystalline regions, reducing water penetration and granule hydration capacity. Swelling factor analysis reveals minimal volume expansion in ASase‐treated starches, with resistance to swelling even at elevated temperatures (B.‐S. Kim et al. 2013; I. Park and Mannaa 2024). In some cases, excessive branch chain entanglement prevents particle breakdown during heating cycles, resulting in loss of trough viscosity (D.‐H. Jung et al. 2022; I. Park and Mannaa 2024). Importantly, the magnitude of these physicochemical alterations varies significantly depending on the botanical source of the starch. This variation is primarily governed by the inherent amylose‐to‐amylopectin ratio and the original crystalline polymorph (A, B, or C type). For instance, waxy starches, which consist almost entirely of amylopectin, provide a remarkably higher concentration of accessible non‐reducing ends for ASase‐mediated elongation compared to normal starches containing amylose. Consequently, highly branched waxy starches typically undergo more extensive structural reorganization, leading to a more pronounced reduction in peak viscosity and swelling power. Furthermore, the initial granular architecture (such as the relatively compact A‐type crystallinity in cereal starches versus the more hydrated B‐type in tuber starches) dictates enzyme accessibility and influences how the elongated branches reorganize into stable B‐type double helices. These restricted swelling characteristics may be beneficial for applications requiring stable product dimension during heating and hydration.

Size‐exclusion chromatography confirms substantial increases in weight‐average molecular weight with corresponding changes in molecular size distribution, supporting macromolecular expansion through chain elongation of AP (R. Wang et al. 2020). Field‐emission scanning electron microscopy demonstrates that modified starches exhibit irregular, particulate structures with rough or stratified surfaces, reflecting the reorganized molecular architecture (D.‐H. Jung et al. 2022; B.‐S. Kim et al. 2013; H. Zhang et al. 2018).

**FIGURE 5:**
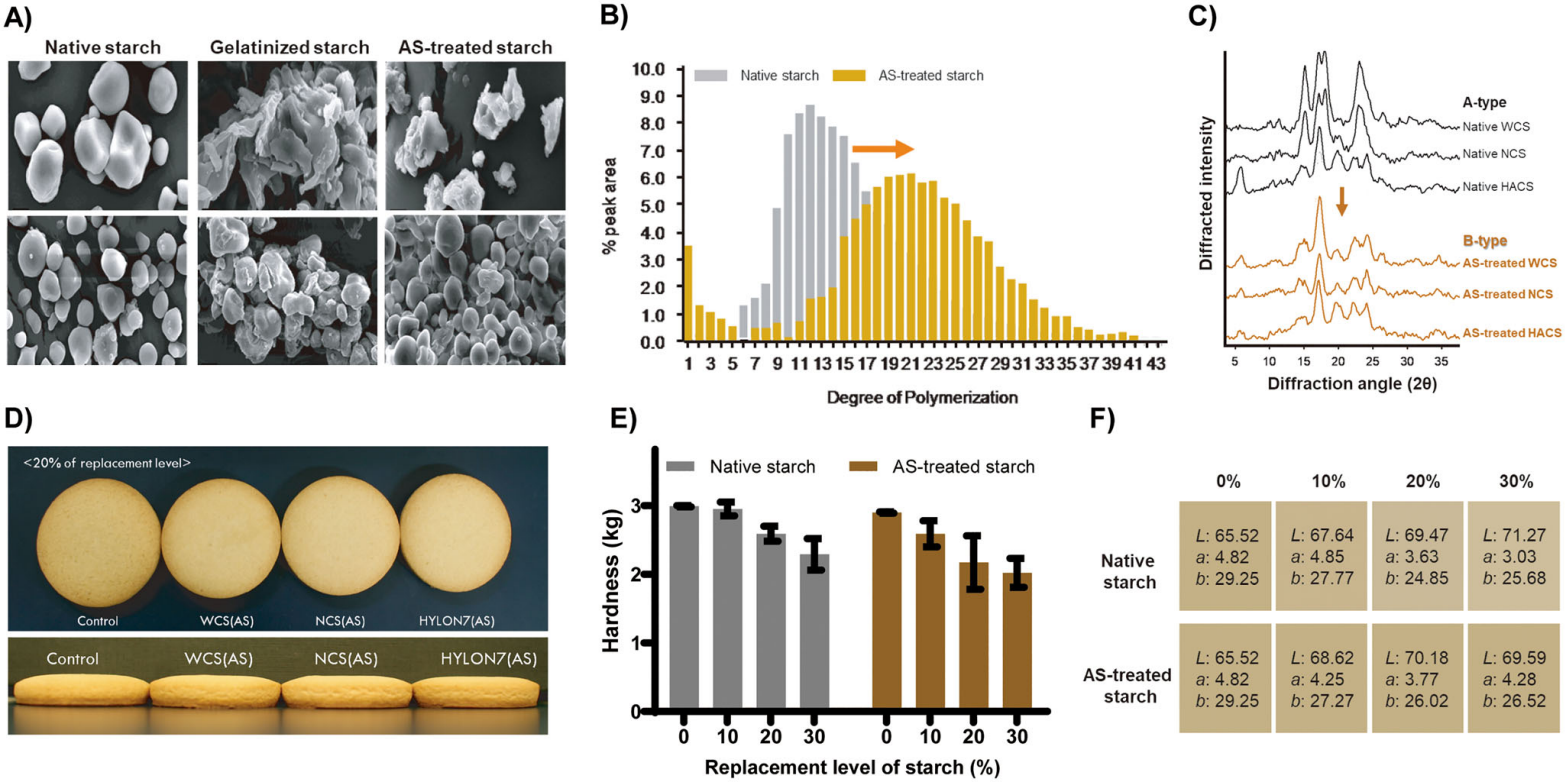
Physicochemical changes in amylosucrase (ASase)‐treated starch and its application in impact on cookie quality. (A) Morphological features observed by scanning electron microscopy, (B) right‐shifted branch‐chain length distribution, and (C) crystalline structure transition from A‐type to B‐type in ASase‐treated starch compared to native starch. (D) Changes in cookie spread ratio and height, (E) hardness profiles, and (F) surface color characteristics of baked cookies prepared with varying levels of ASase‐treated starch.

These altered physicochemical properties of ASase‐treated starch suggest potential for various food applications (Figure 5). Preliminary studies in bakery products have demonstrated that partial flour replacement (10%–30%) with ASase‐modified starch alters dough rheology and cookie spread dynamics (decreased diameter, increased height) (J.‐H. Ryu 2008). This geometric retention during baking is a direct macroscopic consequence of the restricted granule swelling and enhanced thermal stability imparted by the elongated amylopectin branches. Furthermore, the modified formulation significantly increases SDS content (up to 38.7% SDS), suggesting its potential utility in the development of foods with altered glycemic responses, pending further human validation. The modification also results in altered color development (increased *L*‐value, decreased *b*‐value) due to reduced Maillard reaction intensity. Beyond baked goods, the unique physical traits of ASase‐treated starches theoretically align with the technical requirements of other demanding food processes. In general starch applications, restricted swelling is highly valued for preventing granule rupture to maintain structural integrity in canned foods, and for minimizing syneresis to improve freeze–thaw stability in frozen foods. Furthermore, an elevated gelatinization temperature is specifically advantageous during high‐temperature sterilization (e.g., retorting), as it delays premature thickening and maintains optimal heat transfer. Although further empirical studies are required to commercially validate ASase‐treated starches in these specific matrices, their altered physicochemical profiles strongly highlight this industrial potential.

### 6.3 Nutritional Improvements: Digestibility Attenuation and Glycemic Control

ASase treatment substantially increases the proportions of SDS and RS through controlled modification of AP side‐chain distribution. This enzymatic modification elongates external chains, leading to increased proportions of longer chains (DP 25–36 and > 37) while decreasing shorter chains (DP 6–12) (Seo et al. 2020). As discussed in previous sections, this structural rearrangement promotes B‐type crystalline structure formation, creating more stable architectures that are less accessible to digestive enzymes such as α‐amylase and α‐glucosidases (D.‐H. Jung et al. 2022; R. Wang et al. 2023). The enhanced crystalline stability results in significant increases in SDS and RS content. For example, waxy corn starch treated with *Np*AS exhibited a 22.3% increase in RS content, while normal corn starch showed a 20.7% increase (Seo et al. 2020). *Dg*AS‐treated chestnut starches demonstrated even greater improvements, achieving up to 65.0% combined SDS and RS content under optimized conditions (Korompokis et al. 2021).

The increased SDS and RS content alters the digestibility profile of modified starches and attenuates postprandial glucose responses in vitro and in animal models, supporting the potential of these materials as candidates for glycemic management strategies. In vitro digestion kinetics reveal distinct slow digestion phases in ASase‐treated starches, allowing for precise classification based on digestion rates (H. R. Kim, Choi, et al. 2020; I. Park and Mannaa 2024). Animal studies demonstrate significant reductions in blood glucose levels following consumption of ASase‐treated starches compared to native starches (I. Park and Mannaa 2024). These modified digestibility characteristics suggest potential applications in low‐glycemic index food development, though further clinical studies are needed to validate efficacy in human populations.

RS fractions that escape digestion in the small intestine serve as fermentation substrates for colonic bacteria, potentially contributing to gut microbiota modulation and short‐chain fatty acid (SCFA) production. Studies have broadly indicated that fermentation of enzymatically structured starches generates SCFAs such as butyrate, propionate, and acetate, thereby exhibiting prebiotic potential and modulating gut microbiome composition (Miao and BeMiller 2023). While these preliminary results are promising, it is important to distinguish between different levels of evidence. Although current findings derived primarily from in vitro and animal models strongly support the mechanistic link between structural rearrangement mediated by ASase and potential health benefits, these results must be interpreted with caution. Therefore, rigorous human clinical trials are essential to substantiate these health claims regarding glycemic control and prebiotic effects in human populations. The combination of altered digestibility and potential prebiotic effects positions starches modified by ASase as ingredients worthy of continued research for functional food applications.

Starch modification via ASase represents the most mature industrial application due to the relative ease of product recovery from the reaction matrix. However, the operational ceiling is dictated by the high viscosity of concentrated starch slurries, which severely limits mass transfer and volumetric productivity. Addressing this physical constraint is essential for industrial readiness. Future strategies should prioritize the development of continuous reaction systems (e.g., immobilized enzyme reactors) to overcome batch processing limitations and ensure consistent product quality across large‐scale production.

## 7 Biocatalytic Synthesis of Bioactive Glycosides for Nutraceutical and Cosmeceutical Applications

Phenolic and flavonoid substances, which are secondary metabolites of plants, are functional food ingredients due to their potent antioxidant effects (S. Chen et al. 2023). However, the stability and water solubility of these compounds present significant challenges for their industrial application (Tang et al. 2025). Naturally occurring glycosides predominantly feature β‐glycosidic bonds, which often results in reduced bioavailability (Kumar and Pandey 2013). ASases are non‐Leloir glycosyltransferases that catalyze the transfer of glucose from sucrose to various acceptor molecules, forming α‐glycosidic linkages (Seo et al. 2020). The α‐glycosides produced by ASases exhibit distinct functional advantages depending on their specific structural conformations. For instance, some ASase‐derived α‐glycosides demonstrate significantly enhanced chemical stability and water solubility compared to their parent aglycones or β‐glycoside counterparts (Moon et al. 2021). Conversely, recent studies have revealed that even when an α‐glycoside (e.g., luteolin 4′‐O‐α‐glucoside) exhibits lower water solubility and digestive stability than its β‐anomer, it can still demonstrate profoundly superior in vitro intracellular bioactivity by actively upregulating cellular antioxidant enzyme systems (Y. S. Jung et al. 2025). However, it is crucial to note that such enhanced in vitro solubility or cellular bioactivity does not automatically guarantee improved in vivo bioavailability, clinical efficacy, or systemic safety. Comprehensive pharmacokinetic profiling and rigorous toxicological evaluations are strictly required to bridge the gap between these in vitro functional improvements and actual physiological outcomes. This section focuses on the biosynthesis of α‐glycosides by ASases and their potential applications in pharmaceutical and cosmetic industries.

**FIGURE 6:**
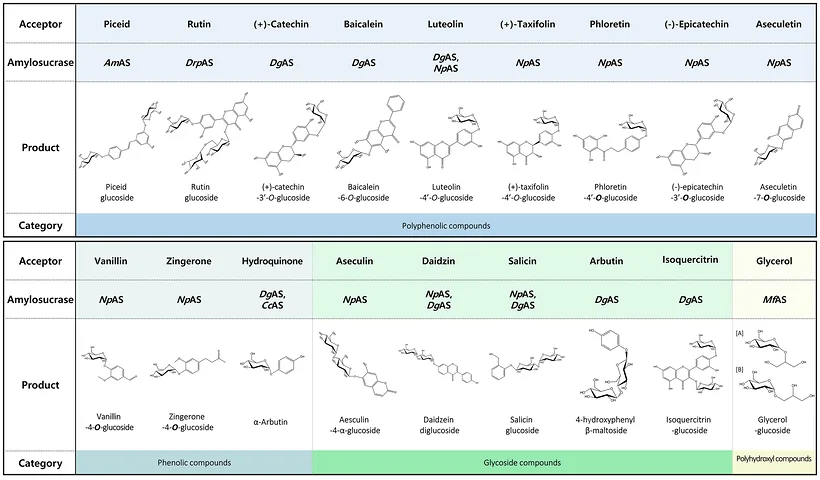
Comprehensive summary of amylosucrase (ASase)‐mediated transglycosylation reactions. The tabular diagram systematically categorizes the diverse acceptor molecules—including polyphenolic, phenolic, and poly‐hydroxyl compounds, as well as existing glycosidic compounds—and their corresponding synthesized α‐glycoside products. ASases efficiently catalyze the transfer of glucose from the donor (sucrose) to these diverse acceptors to produce structurally modified functional glycosides.

ASases demonstrate versatility in transglycosylation reactions, accepting diverse substrates including phenolic compounds (hydroquinone, vanillin), polyphenolic compounds (luteolin, baicalein, catechin derivatives), existing glycoside compounds (arbutin, salicin, daidzin), and poly‐hydroxyl compounds (glycerol, isoquercitrin) (Seo et al. 2020) (Figure 6). Different ASases exhibit varying substrate specificities: *Np*AS shows high activity toward phenolic compounds and certain flavonoids, while *Dg*AS demonstrates superior performance with polyphenolic substrates and glycoside acceptors. The use of sucrose as a cost‐effective alternative to nucleotide sugars, combined with the formation of stable α‐glycosidic linkages, makes ASases attractive biocatalysts for industrial glycoside production.

### 7.1 Glycosylation Strategies for Enhancing Solubility and Stability

In the cosmetic industry, depigmentation and antioxidant compounds represent high‐value ingredients with significant market demand (H. Y. Choi, Lee, et al. 2024). Arbutin (hydroquinone β‐glucoside, β‐arbutin) is a widely used skin lightening agent that functions through tyrosinase inhibition (Seo, Jung, Lee, et al. 2012). ASase‐catalyzed glycosylation produces α‐arbutin (hydroquinone α‐glucoside), which exhibits superior tyrosinase inhibitory activity compared to β‐arbutin (Seo, Jung, Ha, et al. 2012). During α‐arbutin biosynthesis, hydroquinone is susceptible to oxidation, which can reduce conversion efficiency. The addition of ascorbic acid as an antioxidant overcomes this limitation, with *Dg*AS achieving 90% conversion at a 10:1 sucrose to hydroquinone ratio (Seo, Jung, Ha, et al. 2012). Subsequently, several ASases capable of efficient α‐arbutin production without requiring antioxidants have been identified and optimized. *Cc*AS (ASase from *C. carboniz* T26) demonstrated 44% yield within 2 h without ascorbic acid addition (S. Yu et al. 2018), while ASase‐met (ASase gene from the hot spring metagenome) achieved 70% conversion at 30°C, pH 6.0 for 24 h (Agarwal et al. 2021). *Sa*AS (ASase from *Salinispirillum* sp. LH10‐3‐1) showed a higher yield of 60.3% of α‐arbutin than *Cc*AS under identical reaction conditions of raw materials and enzymes as the α‐arbutin biosynthesis conditions of *Cc*AS (J. Li et al. 2023). C. Yang, Fan, et al. (2019) showed that the recombinant *E. coli* JM109 harboring *Xc*AS (ASase from *X. campestris* pv. *campestris* ATCC 33913) achieved α‐arbutin production yield and conversion rate of 60.9 g/L and 95.5%, respectively, by applying bioconversion condition optimization and fed‐batch strategy. While ASases demonstrate remarkable versatility in glycoside synthesis, the reported conversion yields vary significantly depending on the enzyme source and reaction conditions. As observed in α‐arbutin production, yields range broadly from 44% (using *Cc*AS without antioxidants) to 95.5% (using recombinant *Xc*AS). This wide variance is primarily governed by the thermodynamic equilibrium of the transglycosylation reaction, which is highly sensitive to the initial sucrose‐to‐acceptor ratio, the suppression of spontaneous acceptor oxidation (e.g., through ascorbic acid addition), and strategic fed‐batch feeding to prevent product inhibition. Furthermore, the inherent regioselectivity and active‐site topology of different microbial ASases dictate their binding affinity for specific acceptors, highlighting the necessity of combining rational enzyme engineering with optimized bioprocess parameters to achieve commercially viable glycoside yields.

ASases have also been applied to enhance the stability and bioavailability of antioxidant compounds for cosmetic applications. Oxidative stress caused by UV radiation and excess reactive oxygen species (ROS) is a major factor in certain skin damage, including premature aging and skin cancer, making phenolic compounds, which are potent antioxidants, important functional ingredients for cosmetics (J. Chen et al. 2021). Resveratrol (trans‐3,5,4′‐trihydroxystilbene), a potent antioxidant with melanin synthesis inhibitory activity, suffer from poor water solubility and chemical instability (S. Kim et al. 2025). *Dg*AS‐mediated glucosylation produces α‐piceid isomers (resveratrol‐4′‐*O*‐α‐glucoside and resveratrol‐3‐*O*‐α‐glucoside) with conversion rates of 38.7% and 6.8%, respectively, under optimized conditions (10 mM sucrose, 5 mM resveratrol, 20% DMSO) (Moon et al. 2021). The α‐piceid isomers exhibit improved physicochemical properties, including 3.6‐fold and 13.5‐fold higher water solubility compared to β‐piceid (resveratrol‐3‐*O*‐β‐glucoside) and resveratrol, respectively, along with enhanced antioxidant capacity. α‐piceid isomers showed superior cellular uptake in B16F10 cells and more effective melanin synthesis inhibition compared to β‐piceid (Moon et al. 2021).

### 7.2 Substrate Specificity and Regioselectivity in Flavonoid Glycosylation

ASases exhibit high substrate specificity toward flavonoid acceptor molecules, with glycosylation patterns highly depending on structural features and hydroxyl group positioning. Screening study with nine flavone compounds revealed that *Dg*AS selectively catalyzed transglycosylation for substrates containing hydroxyl groups at the 4′‐OH position of the B ring or the 6‐OH position of the A ring (Jang et al. 2018). Luteolin (3',4',5,7‐tetrahydroxyflavone) achieved 86% production yield, while apigenin showed only 20% yield despite their identical backbone structures. Site‐specific study demonstrated that *Dg*AS exhibits strong positional discrimination, with 3‐OH and 7‐OH positions being resistant to transglycosylation while 6‐OH and 4′‐OH positions serve as highly reactive sites (Rha et al. 2019). Kinetic experiments revealed 54‐fold higher catalytic efficiency toward the 6‐OH position compared to the 4′‐OH position, exemplified by baicalein (5,6,7‐trihydroxyflavone) glycosylation producing baicalein‐6‐*O*‐α‐glucoside with 59.1% yield and substantially enhanced water solubility (K. H. Kim, Park, et al. 2014; Rha et al. 2019). The acceptor specificity extends across different flavonoid classes, with isoflavones representing an important substrate category. Studies on soybean isoflavone aglycones revealed conversion yields in the order: genistein > daidzein > glycitein, with glucose attachment preferentially occurring at C‐7 or C‐4′ positions through α‐glycosidic bonds (Y. S. Jung et al. 2023; Y. S. Jung et al. 2020). Notably, products with glucosyl moieties at the C‐4′ position exhibited superior water solubility compared to their C‐7 counterparts, while sequential glycosylation produced polyglucosylated derivatives with dramatically enhanced solubility, as exemplified by genistein 7,4′‐*O*‐α‐diglucoside showed 2459‐fold higher water solubility than parent genistein (Y. S. Jung et al. 2023). These structure–activity relationships demonstrate that acceptor specificity is governed by both the spatial arrangement of hydroxyl groups and the overall flavonoid scaffold architecture.

Different ASases exhibit distinct substrate specificities with important implications for pharmaceutical applications, particularly among *Deinococcus* species variants. ASase from *D. wulumuqiensis* (*Dw*AS) demonstrated superior performance in naringenin α‐glucoside synthesis, achieving a 21.3% biotransformation rate compared to 7.1%–16.2% for other *Deinococcus* ASases (S.‐J. Yu et al. 2024). Molecular analysis revealed that the 217th valine in *Dw*AS corresponds to the 221st isoleucine in *Dg*AS, where the isoleucine residue creates steric hindrance that prevents naringenin from accessing the active site. The reciprocal mutations (*Dw*AS‐V217I and *Dg*AS‐I221V) confirmed this amino acid position's critical role, with the *Dg*AS I221V variant showing twofold higher naringenin α‐glucoside production through increased active site accessibility (S.‐J. Ryu et al. 2024). *Dd*AS demonstrated distinctive product specificity by synthesizing myricetin 4′‐*O*‐4″,6″‐tri‐*O*‐α‐D‐glucopyranoside, a novel branched triglucoside structure that achieved enhanced solubility properties (Im et al. 2024). ASase from *D. planocerae* (*Dpl*AS) exhibited remarkable substrate specificity, predominantly producing naringenin‐4′‐*O*‐α‐d‐maltopyranoside rather than the typical monoglucosides generated by other ASases, demonstrating its unique transglycosylation mechanism that favors maltoside formation over conventional glucoside products (Kang et al. 2023).

Reaction conditions significantly influence enzyme specificity and product formation patterns. *Dg*AS demonstrated the ability to produce different quercetin glycosides depending on pH and substrate concentration, with quercetin 4′‐*O*‐α‐D‐isomaltoside (QG2′) formed predominantly under acidic conditions (pH 5.0) and high sucrose concentrations (1000–1500 mM), while quercetin glucoside (QG1) was generated as an intermediate under neutral conditions (Rha et al. 2020). Kinetic evaluations indicated a 3.9‐fold higher overall performance (*k*_cat_/*K_m_*) for QG2′ generation at pH 5 compared to pH 7, demonstrating controllable product specificity through reaction optimization. In addition, solvent systems significantly impact glycosylation efficiency, with DMSO addition enhancing substrate solubility and enabling higher conversion yields for hydrophobic flavonoids, while donor‐to‐acceptor ratios critically determine product distribution between mono‐ and polyglucosylated derivatives (Y. S. Jung et al. 2020; Moon et al. 2021).

These ASase‐mediated α‐glycoside formations have resulted in significant pharmaceutical improvements. Naringenin α‐glucoside produced by *Dw*AS exhibited enhanced α‐glucosidase inhibitory activity compared to the parent naringenin, indicating potential antidiabetic applications (S.‐J. Yu et al. 2024). Baicalein‐6‐α‐glucoside synthesized by *Dg*AS showed 26.3 times greater solubility than baicalein, enhanced chemical stability in biological media, and demonstrated anti‐inflammatory activity by decreasing nitric oxide production in LPS‐treated RAW 264.7 cells (K. H. Kim, Park, et al. 2014). The synthesis of 13 different α‐linked glucosyl derivatives from soybean isoflavone aglycones by *Dg*AS revealed that products with glucosyl moieties attached to C‐4′ positions exhibited superior water solubility compared to their C‐7 or β‐linkage counterparts, providing highly favorable physicochemical profiles that may potentially improve their formulation characteristics and subsequent physiological absorption (Y. S. Jung et al. 2023).

The application of ASase for bioactive glycoside synthesis is currently in a developing phase, characterized by challenges in substrate solubility and product separation. Unlike starch modification, the modification of hydrophobic flavonoids often requires co‐solvents or biphasic systems, which adds complexity to regulatory approval and downstream purification. For these high‐value ingredients to reach industrial readiness, the field requires a paradigm shift towards green solvent‐free reaction media and the development of GRAS‐certified host strains that can enhance the solubility and stability of aglycones, thereby reducing the dependency on expensive downstream purification.

## 8 Industrial Scale‐Up: Current Commercial Status and Prospective Engineering Strategies

Large‐scale commercialization of ASase reactions requires cost‐effective enzyme production and operational stability. In addition, food‐grade commercial applications mandate the utilization of microbial hosts that are Generally Recognized as Safe (GRAS) to mitigate the inherent endotoxin contamination risks associated with *E. coli* systems. (K. Li et al. 2025; Olempska‐Beer et al. 2006). Recombinant expression platforms utilizing *Corynebacterium glutamicum* and *Bacillus subtilis* provide highly suitable alternative hosts for food‐grade ASase manufacturing (Chin et al. 2020; C. Wang et al. 2024). Comparative structural analyses revealed that the specific microbial expression host directly influences the physical properties of the recombinant enzyme (Chin et al. 2020; Suleimanova et al. 2025). Purified *Dg*AS expressed in *C. glutamicum* exhibited an altered thermal profile with a lower optimal temperature compared to the identical enzyme expressed in *E. coli* (Chin et al. 2020). Circular dichroism spectroscopy attributed this macroscopic thermal variation to a distinct reduction in the strand‐to‐helix ratio within the secondary structure of the *Corynebacterium*‐derived ASase. This host‐dependent structural modification paradoxically reduced the innate polymerization activity while increasing the specific transglycosylation efficiency toward luteolin by 10% (Chin et al. 2020). Engineered *Bacillus* strains similarly achieved the extracellular production of various ASases to synthesize novel sweeteners like turanose and modify complex isoflavones (E.‐R. Kim et al. 2019; C. Wang et al. 2024). Targeted optimization of the twin‐arginine translocation (TAT) pathway in *B. licheniformis* recently enabled this high‐yield extracellular secretion of fully active ASase (C. Wang et al. 2024). Subsequent biocatalytic reactions utilizing these secreted enzymes achieved exceptional conversion rates, significantly enhancing the aqueous solubility of modified soybean isoflavones and delivering remarkable turanose production yields (E.‐R. Kim et al. 2019; C. Wang et al. 2024). These industrially optimized Gram‐positive cell factories frequently enable the direct extracellular secretion of the synthesized enzymes. Secretory production strategies dramatically reduce overall downstream purification costs by completely bypassing complex cell disruption and intracellular extraction steps.

Enzyme immobilization technologies provide a highly effective engineering solution to overcome the economic barriers of single‐use batch reactions by enabling continuous biocatalyst reuse (Sheldon and van Pelt 2013). Covalent attachment to solid supports, including functionalized mesoporous silica and epoxy‐activated acrylic beads, represents the most extensively investigated immobilization strategy for ASases (B.‐Y. Choi, Seo, et al. 2024; H. Lee et al. 2018). Recent engineering efforts specifically demonstrated that immobilizing mutant ASases on mesoporous silica supports significantly enhanced enzyme thermostability, extending the catalytic half‐life from 19.58 h to over 50 h at 55°C (B.‐Y. Choi, Seo, et al. 2024). Industrial‐scale applications utilizing *Dg*AS immobilized on commercial epoxy‐activated beads successfully achieved the sustainable mass production of highly valuable α‐arbutin (H. Lee et al. 2018). This specific immobilized biocatalyst maintained an exceptional 85%–90% target product yield over 35 consecutive reaction cycles, demonstrating significant operational stability and technical feasibility for extended processing. Non‐covalent immobilization approaches utilizing pH‐dependent autoprecipitating polymers, such as Eudragit L100, similarly provide a highly efficient and reversible carrier system for ASases (R. Wang et al. 2011). These smart polymeric carriers enabled the repeated continuous production of linear α‐1,4‐glucans while maintaining robust stability indices across multiple operational cycles. Alternatively, cross‐linked enzyme aggregates (CLEAs) concurrently provide a highly efficient carrier‐free immobilization alternative for complex enzymatic bioprocesses (Sheldon 2011). Advanced co‐immobilization strategies integrating ASases with complementary biocatalysts into combined CLEAs (combi‐CLEAs) successfully facilitated sophisticated one‐pot cascade reactions (M.‐Q. Xu et al. 2018). Tri‐enzymatic combi‐CLEAs comprising ASase, maltooligosyltrehalose synthase, and maltooligosyltrehalose trehalohydrolase explicitly demonstrated this capability by directly converting inexpensive sucrose into high‐value trehalose within a single reaction vessel (D.‐H. Jung et al. 2013). These multi‐enzyme macrostructures effectively eliminated intermediate purification steps and dramatically maximized overall conversion efficiencies during the simultaneous synthesis of customized carbohydrates.

The primary enzymatic cleavage of highly concentrated sucrose substrates by ASase rapidly liberates substantial quantities of free fructose (Seo et al. 2020). This continuously accumulating fructose subsequently acts as a highly preferred acceptor molecule, directing the catalytic flux toward the synthesis of sucrose isomers, such as turanose and trehalulose. However, the high viscosity of concentrated sucrose solutions restricts molecular diffusion and hydrodynamic mixing during standard batch operations (Quintas et al. 2006). Conventional stirred‐tank reactors (STRs) consequently exhibit pronounced mass transfer limitations under these viscous conditions. Therefore, transitioning toward continuous packed‐bed reactor (PBR) configurations is proposed as an engineering solution to mitigate these hydrodynamic bottlenecks. Similar biocatalytic systems utilizing concentrated sucrose as a primary substrate, such as immobilized sucrose isomerases (SIase), provide proof‐of‐concept for this specific reactor scale‐up strategy (Jing et al. 2024). Continuous PBR systems processing extreme sucrose concentrations as high as 600 g/L successfully maintained exceptional catalytic stability and high conversion efficiencies into specific sucrose isomers for extended operational periods (Lina et al. 2002). The conceptual application of these continuous flow architectures to immobilized ASase systems would inherently generate complex saccharide mixtures comprising unreacted sucrose, abundant fructose byproducts, various structural disaccharide isomers, and short‐chain maltooligosaccharides (B.‐Y. Choi, Seo, et al. 2024; Jun et al. 2022; Seo et al. 2020). SMB chromatography currently serves as the most effective industrial methodology for resolving analogous physiochemically similar monomeric, dimeric, and oligomeric sugars continuously at a massive scale, making it a highly promising prospective strategy for future ASase bioprocesses (Azevedo and Rodrigues 2001; Kruschitz and Nidetzky 2020). Although large‐scale SMB applications for specific ASase reaction mixtures have not yet been explicitly reported, analogous enzymatic bioprocesses clearly demonstrate the technical feasibility of this downstream separation strategy. Previous studies have successfully utilized continuous SMB chromatography to fractionate these complex enzymatic broths (Beom et al. 1992; J. Zhang et al. 2025). SMB applications have separated highly similar sucrose isomers, specifically isomaltulose and trehalulose, directly from residual sucrose and monosaccharides, routinely achieving target product purities exceeding 95% with concurrent recovery rates above 90% (Kishihara et al. 1989). Moreover, other reports have successfully validated the separation of short‐chain oligosaccharides, such as fructooligosaccharides (FOS) and specific maltooligosaccharides, from complex carbohydrate mixtures with comparable purities and recovery yields (Vaňková and Polakovič 2012). Despite the theoretical feasibility of continuous PBR and SMB chromatography—as successfully demonstrated in analogous sucrose isomerase systems—the direct industrial application of these technologies to ASase bioprocesses currently faces significant empirical limitations. A major technical bottleneck is the exceptionally high viscosity of concentrated sucrose substrates required for ASase reactions, which severely restricts mass transfer and limits the operational lifespan of immobilized enzyme columns. Furthermore, there is a lack of direct pilot‐scale empirical data validating the long‐term stability and separation efficiency of ASase reaction mixtures in SMB systems. Bridging this gap between laboratory‐scale batch reactions and continuous industrial manufacturing requires urgent engineering efforts to optimize fluid dynamics, develop robust immobilization carriers resistant to high‐viscosity shear stress, and empirically validate downstream SMB separation using actual ASase‐generated carbohydrate mixtures.

Conversely, the downstream processing of high‐molecular‐weight α‐1,4‐glucans necessitates distinctly different physical separation strategies. During extended ASase‐mediated polymerization, long‐chain linear glucans frequently undergo spontaneous crystallization, precipitating directly out of the aqueous reaction broth (Y.‐J. Kim et al. 2024; Potocki‐Veronese et al. 2005; So et al. 2026). Industrial continuous centrifugation effectively harvests these inherently insoluble macroscopic matrices without requiring any chemical additives. However, for the complete recovery of the remaining soluble polymeric fractions, ethanol precipitation serves as the universally established industrial standard (J. Xu et al. 2014). The controlled addition of this specific anti‐solvent rapidly reduces the dielectric constant of the reaction medium, forcefully driving the aggregation and precipitation of the target polysaccharides. Subsequent solid–liquid separation via industrial decanters, followed by large‐scale vacuum drying, ultimately yields highly purified, commercial‐grade glucan powders.

## 9 Critical Challenges and Future Perspectives

**FIGURE 7:**
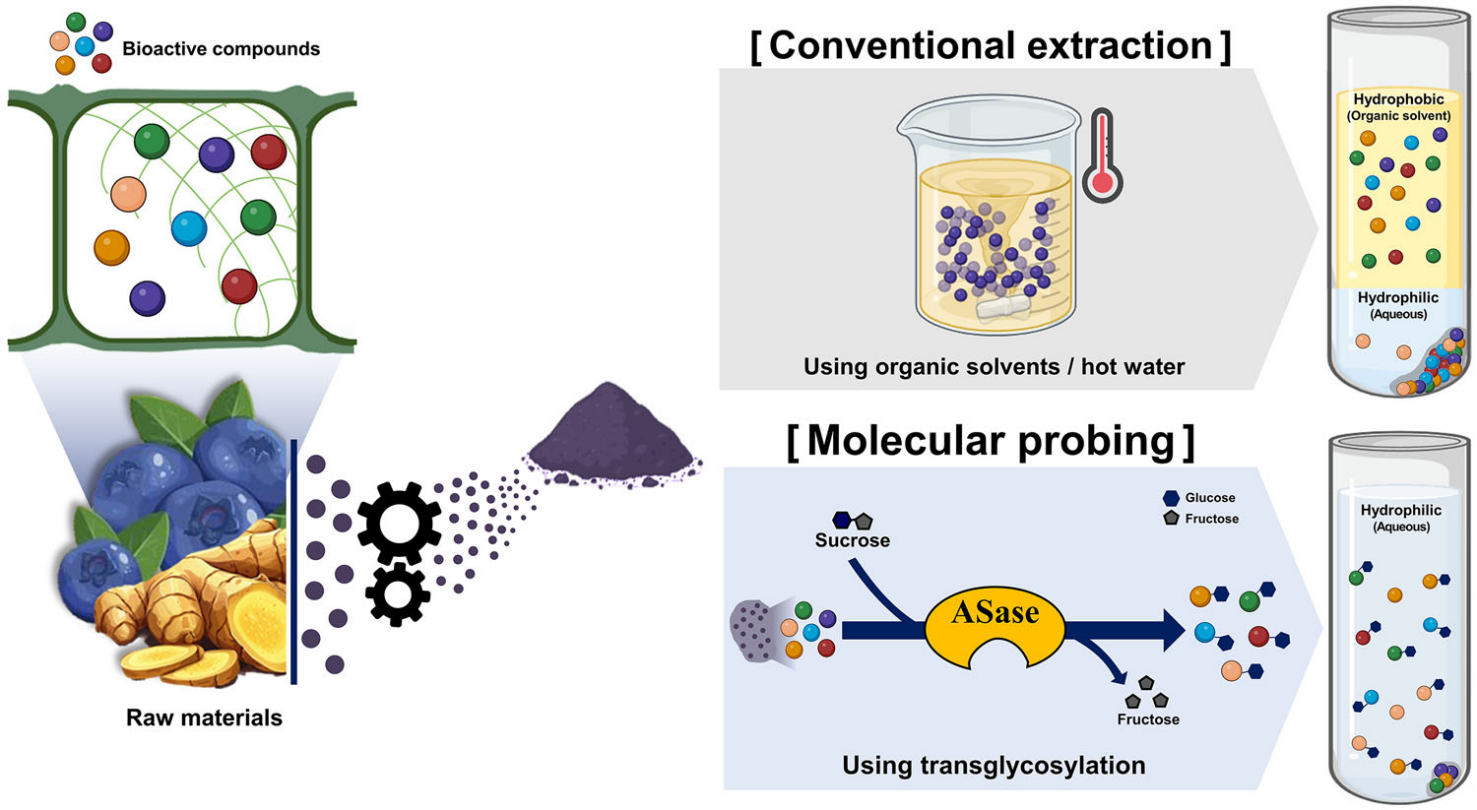
Concept of “molecular probing” (in situ enzymatic extraction) using amylosucrase (ASase). Unlike conventional extraction requiring organic solvents, ASase‐mediated molecular probing directly glycosylates hydrophobic bioactive compounds within the raw material, shifting them into the hydrophilic aqueous phase.

This comprehensive review highlights the versatility of ASase applications across α‐glucan synthesis, starch modification, and pharmaceutical/cosmetic compound development. ASases utilize sucrose as an inexpensive glucose donor to form α‐glycosidic linkages, offering economic advantages over Leloir glycosyltransferases requiring nucleotide sugars. Recent structural studies have elucidated domain organization, oligomeric states, and active site architecture, enabling rational engineering approaches. Key residues influencing substrate recognition have been identified, though the structural basis for broad acceptor promiscuity remains incompletely understood. Process optimization has improved product yields, with reaction conditions (pH, temperature, substrate ratio) significantly affecting product specificity and conversion efficiency. However, several technical challenges hinder the widespread industrial implementation of ASases. The moderate thermostability (30°C–50°C) of native enzymes limits operational versatility, while variable conversion rates, often resulting in suboptimal yields, pose hurdles for process economics. Furthermore, the reliance on organic co‐solvents for hydrophobic substrates impedes efficient downstream processing. Consequently, future research must focus on enhancing enzyme robustness and developing cost‐effective recovery strategies to ensure commercial viability. To directly overcome the reliance on toxic organic solvents, a paradigm‐shifting strategy termed “molecular probing” (or in situ enzymatic extraction) can be proposed for future bioprocessing (Figure 7). Conventional extraction of plant‐derived bioactive compounds heavily depends on organic solvents to recover hydrophobic metabolites. In contrast, the molecular probing approach integrates ASase and sucrose directly into the initial aqueous extraction process of raw plant materials. By simultaneously extracting and reacting, the enzyme actively targets and glycosylates hydrophobic intracellular metabolites in situ. This immediate biotransformation drastically enhances their aqueous solubility, effectively pulling previously unextractable functional compounds into the hydrophilic (aqueous) phase. This innovative strategy not only eliminates the dependency on organic co‐solvents but also radically streamlines the bioprocess by combining extraction and functionalization into a single, eco‐friendly step. To achieve these goals, future research must radically advance both computational enzyme engineering and integrated bioprocessing architectures. The integration of state‐of‐the‐art PLMs offers unprecedented opportunities to rapidly evolve ASase thermostability and manipulate substrate specificity without relying solely on traditional trial‐and‐error directed evolution. Furthermore, predicting the transglycosylation efficiency of complex, hydrophobic acceptors like flavonoids remains a significant hurdle; implementing targeted machine learning models will dramatically accelerate the screening and optimization of these novel target compounds.

From an industrial bioprocessing perspective, overcoming the severe mass transfer and hydrodynamic limitations is essential. Successful translation from laboratory to commercial scale will require shifting toward continuous PBR systems intimately coupled with advanced downstream cascades, specifically SMB chromatography for isomer resolution and automated precipitation systems for continuous glucan recovery. Furthermore, rigorous engineering optimization of fluid dynamics and continuous flow architectures will be required to maximize overall process economics prior to physical scale‐up. In parallel with process optimization, the physiological efficacy and safety profiles of the final products must be rigorously established. For modified starches, clinical validation of glycemic benefits observed in animal studies is needed. Novel α‐glycosides require comprehensive toxicological assessment and regulatory evaluation depending on intended applications. ASase technology has demonstrated technical feasibility across multiple applications. Continued progress in enzyme engineering, process optimization, and economic validation will determine successful translation from laboratory to commercial products.

## Author Contributions

**Dong‐Ho Seo**: writing – original draft, conceptualization. **Byung‐Hoo Lee**: writing – original draft, review and editing, conceptualization. **Sang‐Ho Yoo**: writing – review and editing, supervision, conceptualization, project administration.

## Conflicts of Interest

The authors declare no conflicts of interest.
